# Relationship between the changes in hepatokine levels and metabolic effects after laparoscopic sleeve gastrectomy in severely obese patients

**DOI:** 10.1007/s00595-023-02767-w

**Published:** 2023-11-13

**Authors:** Akira Umemura, Akira Sasaki, Toshinari Takamura, Hiroaki Takayama, Yumie Takeshita, Yosuke Toya, Keisuke Kakisaka, Yutaka Hasegawa, Yasushi Ishigaki

**Affiliations:** 1 Department of Surgery, Iwate Medical University School of Medicine, 2-1-1 Idaidori, Yahaba, Iwate 028-3695 Japan; 2 Department of Endocrinology and Metabolism, Graduate School of Medical Sciences, Kanazawa University, Kanazawa, Ishikawa 920-8640 Japan; 3 Division of Gastroenterology, Department of Internal Medicine, Iwate Medical University School of Medicine, Yahaba, Iwate 028-3695 Japan; 4 Division of Hepatology, Department of Internal Medicine, Iwate Medical University School of Medicine, Yahaba, Iwate 028-3695 Japan; 5 Division of Diabetes, Metabolism and Endocrinology, Department of Internal Medicine, Iwate Medical University School of Medicine, Yahaba, Iwate 028-3695 Japan

**Keywords:** Hepatokine, Selenoprotein P, Leukocyte cell-derived chemotaxin 2, Laparoscopic sleeve gastrectomy, Type 2 diabetes

## Abstract

**Purpose:**

To clarify the relationships between the changes in hepatokines and weight loss, and between these changes and the metabolic effects, and the roles played by these changes, after laparoscopic sleeve gastrectomy (LSG).

**Methods:**

We recruited 25 Japanese patients with severe obesity, who underwent LSG. We measured two hepatokines: selenoprotein P (SeP) and leukocyte cell-derived chemotaxin 2 (LECT2), at the baseline, and then 6 months and 1 year after LSG. Finally, we compared the changes in the hepatokines with the parameters of type 2 diabetes (T2D) and non-alcoholic steatohepatitis (NASH).

**Results:**

Changes in LECT2 were correlated with the percentage of total weight loss (ρ = − 0.499, *P* = 0.024) and the decrease in total fat area (ρ = 0.559, *P* = 0.003). The changes in SeP were correlated with those in hemoglobin A1c (ρ = 0.526, *P* = 0.043) and the insulinogenic index (ρ = 0.638, *P* = 0.010) in T2D patients. In patients with NASH, the LECT2 levels were correlated with liver steatosis (ρ = 0.601).

**Conclusions:**

SeP levels decrease in association with HbA1c reduction, whereas LECT2 levels are associated with reductions in fat mass and NASH scores after LSG. Hepatokines may be involved in the pathology of obesity and its complications.

## Background

The liver plays an essential role in maintaining glucose homeostasis. It is connected anatomically to the pancreas via the portal vein and is primarily affected by the pancreatic hormones. The liver also produces various physiological proteins, such as albumin and coagulation factors. The liver secretes bioactive proteins, termed “hepatokines,” involved in type 2 diabetes (T2D) and non-alcoholic fatty liver disease (NAFLD), including non-alcoholic steatohepatitis (NASH) [1–3]. Among the hepatokines, selenoprotein P (SeP, encoded by *SELENOP* in humans) is upregulated in the diabetic state with hyperglycemia and reduced insulin action and plays a role in diabetic pathology such as insulin resistance in the liver and exercise resistance in the skeletal muscle by eliminating the reactive oxygen species required for signal transduction [4, 5]. Leukocyte cell-derived chemotaxin 2 (LECT2) is upregulated in the livers of people with obesity by sensing liver fat [6, 7]. It may link liver steatosis to inflammation by activating tissue macrophages in mice [8]. However, few reports have demonstrated the relationships between the dynamics of hepatokines and the metabolic effects after metabolic surgery (MS). The present study investigated the dynamics in the blood levels of SeP and LECT2 during weight loss after laparoscopic sleeve gastrectomy (LSG) in patients with severe obesity.

## Methods

### Study design

This research was designed as a retrospective cohort study. Among 124 Japanese patients with severe obesity, who underwent LSG between June, 2008 and December, 2021 at Iwate Medical University Hospital, 25 patients treated between October, 2017 and August, 2021 were enrolled in this study. All participants met the following inclusion criteria for LSG treatment established by Japan’s national health insurance system: age between 18 and 65 years, severe obesity (body mass index [BMI] ≥ 35 kg/m^2^), and a history of at least one comorbidity that resisted medical treatment (hypertension, T2D, dyslipidemia, or obstructive sleep apnea) [9]. The study’s exclusion criteria were a history of alcohol abuse, secondary obesity (drug-induced or related to endocrine diseases), and major psychiatric disorders.

The study protocol was approved by our institution’s ethics committee (H27-42, MH2022-056) and followed the ethical principles stipulated in the Declaration of Helsinki and its subsequent amendments. We obtained informed consent from each participant, and patient anonymity was protected.

## Data collection

A prospective database was used to store the following data from all participants at their baseline, and then 6 and 12 months after LSG: body weight, BMI, percentage total weight loss (%TWL), percentage excess weight loss (%EWL) to evaluate weight-loss effects, hemoglobin A1c (HbA1c), fasting glucose, immunoreactive insulin (IRI), C-peptide, a homeostasis model of assessing insulin resistance (HOMA-IR), a homeostatic model assessment of beta cell function (HOMA-β), the insulinogenic index (II) to evaluate T2D and aspartate aminotransferase (AST), alanine aminotransferase (ALT), platelet count, ferritin, type IV collagen 7S, and hyaluronic acid to evaluate NASH. In this study, we administered a 75 g oral glucose tolerance test to evaluate the participants’ insulin secretion and resistance.

Liver volume (LV), visceral fat area (VFA), and subcutaneous fat area (SFA) were calculated through computed tomography (CT) when laboratory data were collected. These calculations used a 64-row CT (AquilionTM; Toshiba Medical Systems Corporation; Tokyo, Japan). After obtaining a single tomographic slice at the umbilicus level, VFA and SFA were detected and measured using a Hounsfield unit (HU) range for adipose tissue of -150–0 HU, and then calculated and recorded in cm^2^. To measure LV, CT images were downloaded as digital images and copied from medical files to a computer workstation (SYNAPSE VINCENT; Fujifilm; Tokyo, Japan). The LV was recorded in mL.

The participants’ concentrations of serum SeP were measured using a single-particle homogeneous immunoassay. We assessed the full-length SeP serum levels selectively, using two types of SeP monoclonal antibodies. The first type indicates the N-terminal domain of SeP, and the other type indicates the C-terminal domain [10]. Furthermore, patients’ LECT2 plasma levels were measured using the Ab-Match ASSEMBLY Mouse LECT2 kit (CircuLex Mouse LECT2 ELISA Kit, Medical & Biological Laboratories Co., Ltd., Japan).

### Histopathological examination of NASH

All participants underwent intraoperative liver biopsies, because preoperative ultrasound-guided liver biopsies were associated with risks related to the patients’ thick subcutaneous fat tissue. When abdominal wall thickness was over 50 mm, the image resolution, including intrahepatic vessels, deteriorated because of attenuation of the ultrasound; therefore, preoperative ultrasound-guided liver biopsy for patients with severe obesity was usually avoided. The specimens were formalin-fixed and stained with hematoxylin–eosin, silver reticulin, and Masson trichrome to check for liver fibrosis. The histopathological findings were recorded using steatosis percentages, Matteoni classification, Brunt classification, and NAFLD activity scores [11–13]. Patients with NASH underwent protocol liver biopsies 6 months and 1 year after their LSGs.

### Statistical analysis

This study’s data were recorded as numbers, percentages for the categorical variables, and means ± standard deviations for the continuous variables. Statistical analysis was performed using chi-square tests for the categorical variables and student’s t-tests or Mann–Whitney U tests for continuous variables. The correlations between hepatokines and weight loss, as well as the metabolic effects, were analyzed using Spearman’s correlation coefficient. We considered *p *values of less than 0.05 significant and all reported *p*-values were two-sided. We evaluated how accurately the prevalence of T2D and NASH had been detected by creating receiver operating characteristic (ROC) curves and calculating values for the areas under the curves (AUCs). All statistical analyses were performed using JMP Pro, version 15 (SAS Institute Inc., Cary, NC, USA).

## Results

### Patients’ backgrounds

Table 1 summarizes the clinical characteristics of the 25 patients. The mean initial body weight and BMI were 116.1 kg and 42.1 kg/m^2^, respectively. The obesity-related diseases, T2D and histopathological NASH, were diagnosed from intraoperative liver biopsy in 15 and 17 of the enrolled patients, respectively.

**Table 1:** Clinical characteristics of the enrolled patients

Variables	
Age (years)	41.8 ± 10.2
Gender (Male / Female)	16/9
Initial body weight (kg)	116.9 ± 17.0
Initial BMI (kg/m^2^)	42.1 ± 5.1
Obesity-related disease (*n*, %) T2D Histopathological NASH Hypertension Dyslipidemia Hyperuricemia Obstructive sleep apnea	15, 60.0 17, 68.0 22, 88.0 18, 72.0 16, 64.0 24, 96.0

*BMI* body mass index, *T2D* type 2 diabetes, *NASH* non-alcoholic steatohepatitis

### Weight loss and the metabolic effects of LSG

Table 2 shows the weight loss and metabolic effects of the enrolled patients after LSG. The mean body weight loss and BMI loss 1 year after LSG were 34.6 kg (*p* < 0.001) and 12.0 kg/m^2^ (*p* < 0.001), respectively. Meanwhile, their mean %TWL and %EWL were 28.3% and 60.4%, respectively. Concerning fat tissue volume, the VFA and SFA decreased significantly post-LSG (*p* < 0.001).

**Table 2:** Weight loss effects and improvement in metabolic parameters

Variables	Baseline	6 months after LSG	1 year after LSG	*p* value
Body weight (kg)	116.9 ± 17.0	86.0 ± 13.3	82.3 ± 13.7	< 0.001
BMI (kg/m^2^)	42.1 ± 5.1	30.8 ± 3.6	30.1 ± 4.1	< 0.001
%TWL (%)	–	26.1 ± 7.5	28.3 ± 7.9	–
%EWL (%)	–	56.1 ± 13.9	60.4 ± 15.1	–
HbA1c (%)	7.7 ± 1.7	5.5 ± 0.4	5.4 ± 0.3	< 0.001
HOMA-IR (no unit)	6.9 ± 6.4	1.8 ± 1.2	1.8 ± 1.1	< 0.001
HOMA-β (no unit)	129.5 ± 78.3	109.4 ± 50.4	133.4 ± 72.6	0.848
II (no unit)	3.7 ± 4.6	1.1 ± 0.5	1.2 ± 0.6	< 0.001
AST (U/L)	42.0 ± 32.4	16.7 ± 4.2	16.8 ± 4.6	< 0.001
ALT (U/L)	67.1 ± 56.3	17.6 ± 4.2	16.7 ± 7.0	< 0.001
Platelet count (× 10^4^/μL)	25.6 ± 5.4	23.3 ± 5.0	22.8 ± 6.2	0.134
Ferritin (ng/mL)	163.8 ± 142.2	98.1 ± 73.4	70.4 ± 59.0	0.012
Type IV collagen 7S (ng/mL)	4.8 ± 1.2	4.8 ± 0.9	4.5 ± 0.7	0.452
Hyaluronic acid (ng/mL)	21.4 ± 12.2	30.9 ± 19.1	32.7 ± 19.0	0.022
LV (mL)	2138.0 ± 438.2	1600.4 ± 270.2	1567.7 ± 213.5	< 0.001
VFA (cm^2^)	241.6 ± 58.9	137.7 ± 70.5	118.2 ± 64.0	< 0.001
SFA (cm^2^)	560.5 ± 143.0	333.6 ± 116.0	307.6 ± 131.7	< 0.001

*BMI* body mass index, *%TWL* percentage total weight loss, *%EWL* percentage excess weight loss, *HbA1c* hemoglobin A1c, *HOMA-IR* homeostasis model assessment-insulin resistance, *HOMA-β* homeostatic model assessment beta cell function, *II* insulinogenic index, *AST* aspartate aminotransferase, *ALT* alanine aminotransferase, *LV* liver volume, *VFA* visceral fat area, *SFA* subcutaneous fat area

The metabolic parameters of T2D, HbA1c, HOMA-IR, and II had improved significantly by 1 year after LSG in 15 patients with T2D (Table 3). Moreover, 11 patients (77.3%) had evidence of T2D remission 1 year after LSG. Only four patients were receiving anti-diabetic therapy, and the total number of anti-diabetic drugs that patients were taking dropped from 47 to 5. By 1-year post-LSG, sulfonylurea, thiazolinedione, sodium glucose cotransporter-2 inhibitor, alpha-glucosidase inhibitor, and glucagon-like peptide 1 receptor agonist had been discontinued.

**Table 3:** Metabolic effects and changes in hepatokines in patients with type 2 diabetes (*n* = 15)

Variables	Baseline	6 months after LSG	1 year after LSG	*p* value
Age (years)	41.7 ± 10.9	–	–	–
BMI (kg/m^2^)	42.3 ± 5.4	30.8 ± 3.6	30.1 ± 4.1	< 0.001
C-peptide (ng/mL)	2.9 ± 1.2	1.5 ± 0.7	1.3 ± 0.6	< 0.001
Duration of T2D (years)	6.1 ± 5.9	–	–	–
ABCD score (points)	5.4 ± 1.9	–	–	–
HbA1c (%)	7.7 ± 1.7	5.5 ± 0.4	5.4 ± 0.3	< 0.001
HOMA-IR (no unit)	6.9 ± 6.4	1.8 ± 1.2	1.8 ± 1.1	< 0.001
HOMA-β (no unit)	129.5 ± 78.3	109.4 ± 50.4	133.4 ± 72.6	0.848
II (no unit)	3.7 ± 4.6	1.1 ± 0.5	1.2 ± 0.6	< 0.001
SeP (μg/mL)	3.7 ± 0.5	3.4 ± 0.6	3.6 ± 0.7	0.445
LECT2 (ng/mL)	41.5 ± 11.2	28.2 ± 13.2	32.3 ± 9.4	0.011
Remission rate of T2D * (n, %)	–	9, 60.0	11, 73.3	–
Administration of anti-diabetic agents (*n*) Metformin Sulfonylurea Thiazolinedione α-glucosidase inhibitor DPP-4 inhibitor SGLT-2 inhibitor GLP-1 agonist Insulin	10 7 3 3 12 5 3 4	5 0 0 0 2 0 0 1	3 0 0 0 1 0 0 1	

*BMI* body mass index, *T2D* type 2 diabetes, *HbA1c* hemoglobin A1c, *HOMA-IR* homeostasis model assessment-insulin resistance, *HOMA-β* homeostatic model assessment beta cell function, *II* insulinogenic index, *SeP* selenoprotein P, *LECT2* leukocyte cell-derived chemotaxin 2: DPP-4 inhibitor, dipeptidyl peptidase 4 inhibitor; SGLT-2 inhibitor, sodium-glucose cotransporter 2 inhibitor, *GLP-1 agonist* glucagon-like peptide 1 receptor agonist

^*^ Remission of T2DM was defined as HbA1c > 6.5% without any anti-diabetic agents

Histopathological NASH was observed in 17 patients and the other patients were diagnosed with non-alcoholic fatty liver (NAFL). The changes in every parameter and the histopathological changes in patients with NASH are shown in Table 4. The metabolic parameters of NASH (AST, ALT, platelet count, and ferritin) improved significantly in all patients with histopathological NASH. However, no significant changes in type IV collagen 7S or hyaluronic acid were observed. Furthermore, complete NASH resolution was achieved in 11 patients 1 year post-LSG, although histopathological NASH persisted for 5 patients. Concerning the changes in histopathological findings, histopathological steatosis, the grade and stage of the Brunt classification, and the total NAFLD activity score improved significantly after LSG.

**Table 4:** Metabolic effects and changes in hepatokines in patients with non-alcoholic steatohepatitis (*n* = 17)

Variables	Baseline	6 months after LSG	1 year after LSG	*p* value
AST (U/L)	47.4 ± 35.9	17.7 ± 4.2	18.0 ± 4.8	< 0.001
ALT (U/L)	76.1 ± 57.1	19.0 ± 8.4	17.6 ± 7.5	< 0.001
Platelet count (× 10^4^/μL)	25.3 ± 5.7	23.2 ± 5.5	22.6 ± 6.2	0.007
Ferritin (ng/mL)	188.2 ± 158.0	93.3 ± 73.8	66.8 ± 60.3	< 0.001
Type IV collagen 7S (ng/mL)	4.9 ± 1.4	4.7 ± 0.7	4.4 ± 0.8	0.124
Hyaluronic acid (ng/mL)	21.1 ± 12.5	26.5 ± 15.2	28.1 ± 17.8	0.744
SeP (μg/mL)	3.7 ± 0.5	3.4 ± 0.7	3.6 ± 0.7	0.682
LECT2 (ng/mL)	41.0 ± 12.0	31.2 ± 12.9	33.2 ± 9.5	0.039
Diagnosis of liver biopsy NASH NAFL Normal liver	17 0 0	7 2 8	5 1 11	–
Histopathological steatosis (%)	24.7 ± 19.7	5.1 ± 3.9	3.7 ± 4.8	< 0.001
Matteoni classification 1 2 3 4	0 0 0 17	3 1 0 3	4 1 0 0	–
Brunt Grade Stage	1.1 ± 0.4 1.4 ± 0.9	0.1 ± 0.3 0.6 ± 1.0	0.1 ± 0.3 0.7 ± 1.0	< 0.001 0.002
NAFLD activity score Steatosis Inflammation Ballooning Total	1.2 ± 0.8 1.1 ± 0.8 0.4 ± 0.6 2.5 ± 1.8	0.2 ± 0.4 0.1 ± 0.4 0.1 ± 0.3 0.4 ± 0.8	0.1 ± 0.4 0.2 ± 0.4 0.0 ± 0.0 0.3 ± 0.6	< 0.001 < 0.001 0.029 < 0.001

*AST* aspartate aminotransferase, *ALT* alanine aminotransferase, *SeP* selenoprotein P, *LECT2* leukocyte cell-derived chemotaxin 2, *NAFLD* non-alcoholic fatty liver disease, *NASH* non-alcoholic steatohepatitis

### Changes in the circulating levels of hepatokines and their relationship with weight loss and metabolic effects

**Fig. 1:**
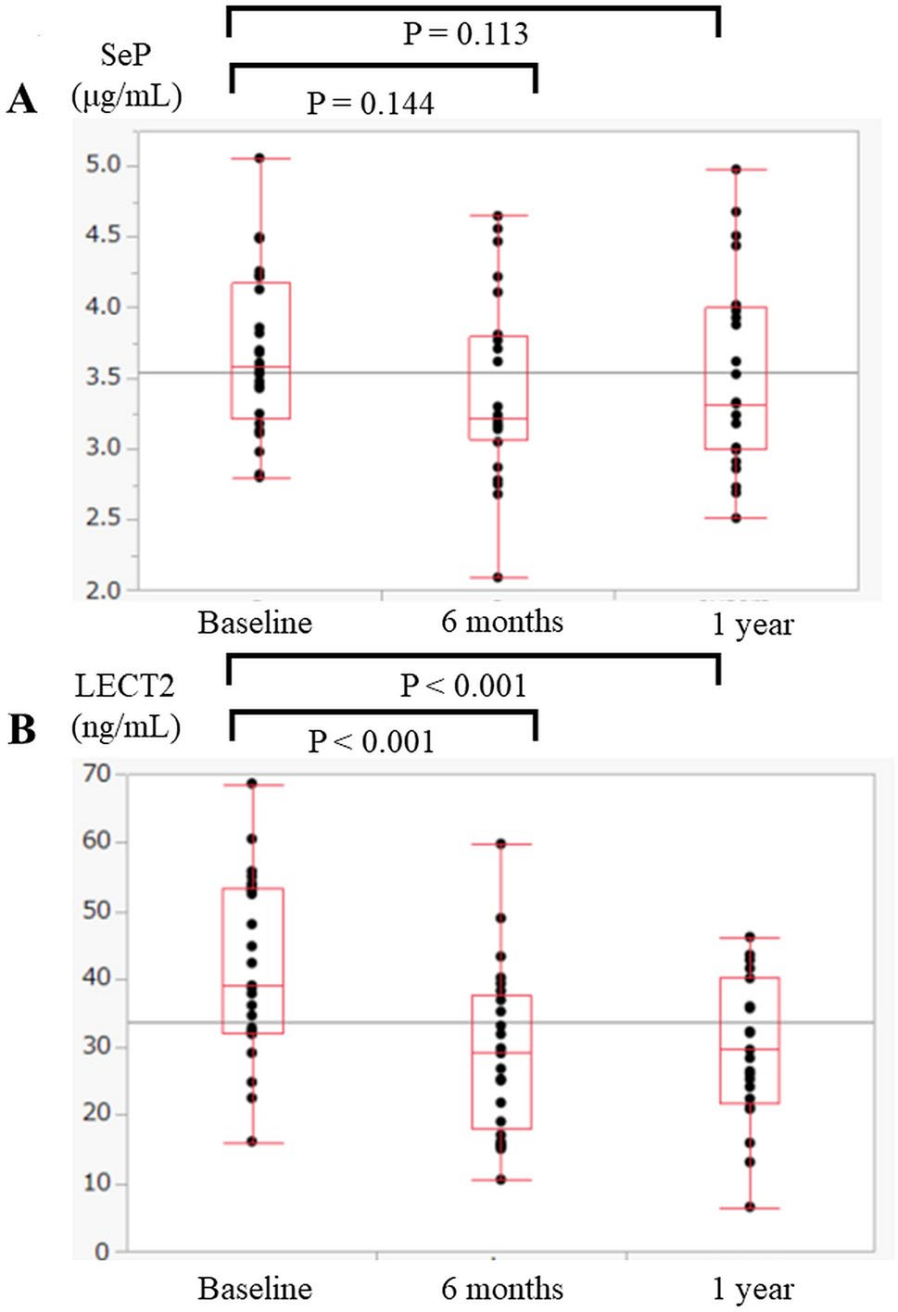
Changes in selenoprotein P (SeP) and leukocyte cell-derived chemotaxin 2 (LECT2). **A** The changes in SeP from baseline to 1 year after laparoscopic sleeve gastrectomy (LSG) were not significant. **B** The serum levels of LECT2 after LSG decreased significantly from the baseline

Figure 1 shows the changes in circulating levels of the hepatokines. The SeP levels had not changed significantly by 6 months (*p* = 0.144) or 1 year (*p* = 0.113) after LSG. In contrast, the LECT2 decreased significantly after LSG (*p* < 0.001). Table 5 shows the correlations between the baseline levels of SeP, LECT2, and the metabolic parameters. Significant correlations were detected between SeP and VFA + SFA (ρ = − 0.522, *p* = 0.045), LECT2 and body weight (ρ = − 0.662, *p* = 0.010), and LECT2 and LV (ρ = − 0.564, *p* = 0.035) at baseline. Furthermore, the correlations between LECT2 and transaminases were relatively high but not significant at baseline. Significant correlations between SeP levels and VFA were observed at 6 months and 1 year after LSG. Moreover, we analyzed the relationships between the changes in SeP and LECT2, and between weight loss and metabolic markers (Table 6). The changes in SeP did not correlate with any weight loss or metabolic parameters, while the changes in LECT2 correlated significantly with certain weight-loss parameters such as changes in %TWL (ρ = − 0.499, *p* = 0.024), VFA (ρ = 0.450, *p* = 0.024), SFA (ρ = 0.446, *p* = 0.025), and VFA + SFA (ρ = 0.559, *p* = 0.003).

**Table 5:** Multiple correlation analysis between all data on hepatokines and metabolic parameters

Variables	Baseline				6 months after LSG				1 year after LSG			
	SeP (μg/mL)		LECT2 (ng/mL)		SeP (μg/mL)		LECT2 (ng/mL)		SeP (μg/mL)		LECT2 (ng/mL)	
	ρ	*p* value	ρ	*p* value	ρ	*p* value	ρ	*p* value	ρ	*p* value	ρ	*p* value
Body weight (kg)	− 0.070	0.811	0.662	0.010	0.330	0.168	0.011	0.964	0.128	0.612	− 0.050	0.843
BMI (kg/m^2^)	− 0.140	0.632	0.425	0.129	0.294	0.221	0.133	0.588	0.170	0.500	0.313	0.206
AST (U/L)	0.140	0.632	0.400	0.156	0.483	0.036	0.154	0.530	0.272	0.274	0.293	0.239
ALT (U/L)	0.172	0.555	0.510	0.062	0.396	0.093	0.029	0.907	− 0.036	0.888	− 0.054	0.833
Ferritin (ng/mL)	0.226	0.438	0.283	0.326	0.274	0.255	− 0.203	0.405	0.112	0.659	− 0.367	0.134
Type IV collagen 7S (ng/mL)	0.117	0.691	0.054	0.854	0.410	0.081	− 0.085	0.729	0.109	0.667	0.095	0.708
Hyaluronic acid (ng/mL)	0.286	0.321	0.306	0.286	0.238	0.327	− 0.194	0.427	0.071	0.781	0.048	0.849
LV (mL)	0.093	0.752	0.564	0.035	0.167	0.487	0.159	0.516	− 0.182	0.470	-0.326	0.187
VFA (cm^2^)	0.135	0.644	0.303	0.291	0.610	0.006	0.372	0.116	0.535	0.022	0.258	0.302
SFA (cm^2^)	− 0.389	0.169	0.058	0.849	0.104	0.673	0.137	0.576	− 0.211	0.402	0.054	0.833
VFA + SFA (cm^2^)	− 0.552	0.045	0.083	0.779	0.335	0.162	0.247	0.309	0.159	0.530	0.431	0.074

*SeP* selenoprotein P, *LECT2* leukocyte cell-derived chemotaxin 2, *BMI* body mass index, *AST* aspartate aminotransferase, *ALT* alanine aminotransferase, *LV* liver volume, *VFA* visceral fat area, *SFA* subcutaneous fat area

**Table 6:** Multiple correlation analysis between changes in hepatokines and weight-loss and metabolic parameters

Variables	ΔSeP (μg/mL)		ΔLECT2 (ng/mL)	
	ρ	*p* value	ρ	*p* value
Δbody weight (kg)	0.015	0.943	0.345	0.090
%TWL (%)	− 0.044	0.983	− 0.499	0.024
%EWL (%)	− 0.249	0.228	− 0.273	0.186
ΔBMI (kg/m^2^)	− 0.006	0.974	0.366	0.071
ΔAST (U/L)	0.084	0.686	0.110	0.599
ΔALT (U/L)	0.061	0.770	0.109	0.603
ΔFerritin (ng/mL)	0.032	0.878	− 0.044	0.832
ΔType IV collagen 7S (ng/mL)	0.093	0.655	0.028	0.891
ΔHyaluronic acid (ng/mL)	0.139	0.505	0.012	0.954
ΔLV (mL)	0.212	0.308	− 0.069	0.741
ΔVFA (cm^2^)	− 0.138	0.509	0.450	0.024
ΔSFA (cm^2^)	− 0.020	0.922	0.446	0.025
ΔVFA + SFA (cm^2^)	− 0.167	0.424	0.559	0.003

*SeP* selenoprotein P, *LECT2* leukocyte cell-derived chemotaxin 2, *%TWL* percentage total weight loss, *%EWL* percentage excess weight loss, *BMI* body mass index, *AST* aspartate aminotransferase, *ALT* alanine aminotransferase, *LV* liver volume, *VFA* visceral fat area, *SFA* subcutaneous fat area

**Fig. 2:**
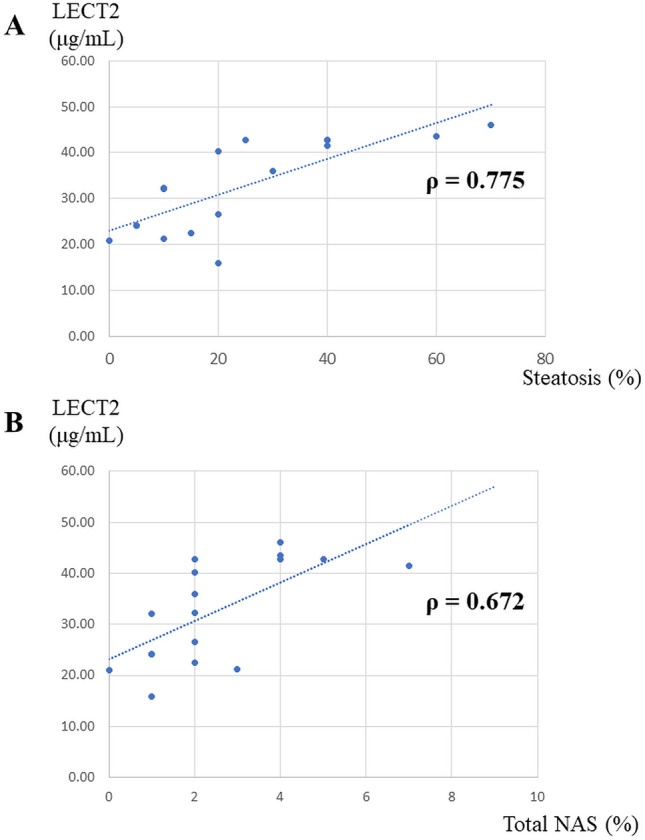
Correlation between the histopathological findings of liver biopsy and the initial leukocyte cell-derived chemotaxin 2 (LECT2) level. **A** The percentage of liver steatosis was correlated with the initial LECT2 level. **B** The total non-alcoholic fatty liver disease (NAFLD) activity score was also correlated with the initial LECT2 level

The same correlation analyses were performed for the patients with T2D (*n* = 15), but no significant correlations were found between the initial levels of SeP and LECT2 and the T2D parameters. Table 7 shows the relationships between the changes in SeP and LECT2 and the T2DM parameters. The changes in SeP correlated significantly with the changes in HbA1c (ρ = 0.526, *p* = 0.043) and II (ρ = 0.638, *p* = 0.010). Simple correlation analyses between the initial LECT2 levels and liver histology revealed that the LECT2 levels correlated with liver steatosis (ρ = 0.775) and the total NAFLD activity score (ρ = 0.672) (Fig. 2).

**Table 7:** Multiple correlation analysis between changes in hepatokines and type 2 diabetes parameters

Variables	ΔSeP (μg/mL)		ΔLECT2 (ng/mL)	
	ρ	*p* value	ρ	*p* value
ΔIRI (μU/mL)	− 0.152	0.635	0.134	0.635
ΔBS (mg/dL)	0.013	0.962	0.001	0.995
ΔHbA1c (%)	0.526	0.043	− 0.051	0.856
ΔHOMA-IR (no unit)	− 0.016	0.592	0.150	0.592
ΔHOMA-β (no unit)	0.201	0.472	0.354	0.195
ΔII (no unit)	0.638	0.010	0.293	0.288

*SeP* selenoprotein P, *LECT2* leukocyte cell-derived chemotaxin 2, *IRI* immunoreactive insulin, *BS* blood sugar, *HbA1c* hemoglobin A1c, *HOMA-IR* homeostasis model assessment-insulin resistance, *HOMA-β* homeostatic model assessment beta cell function, *II* insulinogenic index

### Evaluation of hepatokines as diagnostic tools

**Fig. 3:**
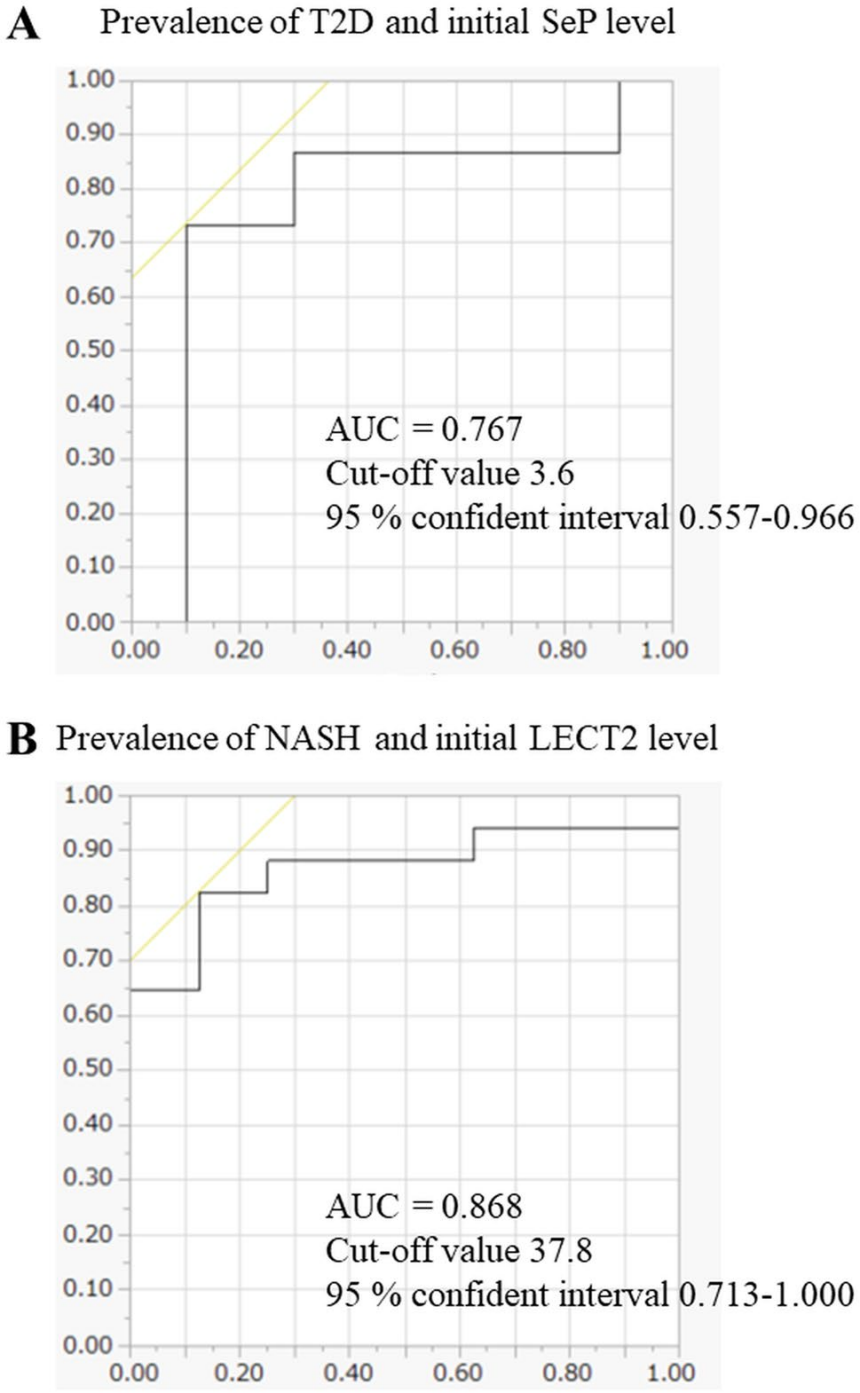
ROC curves of selenoprotein P (SeP) for type 2 diabetes (T2D) and leukocyte cell-derived chemotaxin 2 (LECT2) for non-alcoholic steatohepatitis (NASH). **A** The sensitivity for the prevalence of T2D was 63.3% according to the initial SeP level. **B** The sensitivity for the prevalence of NASH was 69.9% according to the initial LECT2 level

Figure 3 shows the ROC curves between the preoperative hepatokines and T2D and NASH. Initial SeP levels indicated T2D (AUC = 0.767, cut-off value = 3.6 μg/mL, 95% confident interval: 0.557–0.966). Meanwhile, initial LECT2 levels also indicated NASH prevalence (AUC = 0.868, cut-off value = 37.8 ng/mL, 95% confident interval: 0.713–1.000).

## Discussion

In this study, we analyzed changes in hepatokines and the metabolic effects of LSG in Japanese patients with severe obesity. The initial SeP levels correlated negatively with the VFA + SFA, whereas the initial LECT2 levels correlated positively with body weight, LV, histopathological liver steatosis, and the total NAFLD activity score. Furthermore, changes in LECT2 were negatively correlated with %TWL and changes in both SFA and VFA. The changes in SeP were also correlated with the changes in HbA1c and II. Moreover, SeP and LECT2 could predict T2D and NASH, respectively, in the severely obese Japanese patients.

Studies have identified more than 20 hepatokines, some of which play a key role in promoting metabolic syndrome, including T2D and NAFLD. Fetuin-A, the most widely studied hepatokine in humans, acts as a chemoattractant for macrophages, thereby increasing the expression of inflammatory cytokines. These inflammatory cytokines contribute to T2D and NAFLD [14]. Moreover, circulating fetuin-A inhibits glucose uptake by interfering with the translocation activity of glucose transporter type 4 [14]. Angioprotein-like protein 8 (ANGPTL8) is expressed primarily in liver and visceral adipocytes and associated with NASH by promoting various inflammatory pathways [15]. Fibrinogen-like protein 1 is also synthesized mainly in parenchymal hepatocytes and involved in conditions such as liver regeneration, immune activation, glucose and lipid metabolism [16]. In addition to SeP and LECT2, these hepatokines and metabolic disorders are complexly intertwined; therefore, the measurement and activity of hepatokines may help to identify a deeper mechanism of accelerating metabolic disorders in patients with severe obesity.

Metformin is commonly used as an antidiabetic agent and provides clear benefits in promoting glucose metabolism and preventing diabetic complications. It has been shown to act via a mechanism dependent on adenosine-monophosphate-activated protein kinase (AMPK), which includes the suppression of hepatic glucogenesis. We demonstrated previously that metformin suppresses the production of insulin-resistance-inducing SeP by activating AMPK and, subsequently, inactivating FoxO3a in the liver without directly stimulating pancreatic β-cells [17]. Through these mechanisms, metformin administration at the baseline might suppress the elevation of SeP levels; therefore, the true increase in SeP was negated despite the acute insulin resistance caused by severe obesity. In the current study, 10 of the 15 patients with T2D were given metformin at their baseline, and the initial SeP levels were significantly lower in these patients than in the patients not given metformin (3.5 μg/mL vs. 4.1 μg/mL, *p* = 0.023). On the other hand, no significant changes in SeP levels were observed 1 year after LSG. Most patients could stop taking metformin at the 1-year-post-LSG mark, presenting a high remission rate for T2D; thus, the participants’ SeP levels 1 year post-LSG were close to their true value.

The present study illustrated the positive correlation among initial LECT2 levels, body weight, and LV. Additionally, weight loss effects such as changes in %TWL and fat areas also correlated with changes in LECT2. These results suggest that LECT2 is a promising marker for the weight loss effects of MS. We identified a negative correlation only between LECT2 and %TWL, in contrast with the fat areas. This result can be explained by the fact that the body weight loss effect is brought about not only by fat reduction but also by muscle catabolism after LSG. However, we did not evaluate muscle catabolism and skeletal muscle volume in this study. Another clinical study measuring both LECT2 and skeletal muscle volume by bioelectrical impedance analysis is needed to clarify the true relationship between LECT2 and %TWL.

The new concept of metabolic-dysfunction-associated fatty liver disease (MAFLD) has been proposed by international expert panels [18]. Severely obese patients should be diagnosed with MAFLD without imaging modalities or liver biopsies, along with the definition and interpretation of MAFLD. We found previously that about 70% of patients with severe obesity presented with NASH using intraoperative liver biopsies and that the severity of T2D prevalence is closely related to the progression of hepatocyte ballooning and liver fibrosis [19, 20]. Based on these findings, we conclude that NASH in severely obese patients can be automatically included in MAFLD, and it is currently considered a very important targeted MS disease [21]. We reported recently that the hepatic expression of LECT2 is upregulated in association with the inflammatory signature in human liver tissues [8]. Furthermore, the elevation of LECT2 levels activates the pathway from TGF-β-activated kinase 1-binding protein and mitogen-activated protein kinase 4 to the c-Jun N-terminal kinase (JNK) induced by lipopolysaccharides as an adjuvant and shifts residual liver macrophages to the M1-like phenotype, contributing to the development of liver inflammation [22, 23]. Li et al. also demonstrated that thrombospondin-1 (THBS-1) is a reliable biomarker of NASH, advances liver fibrosis, and is closely related to the JNK pathway [23]. These duplicated stimulations of liver macrophages may rapidly accelerate liver inflammation. From the current MAFLD algorithm, patients undergoing MS are automatically diagnosed as having MAFLD from severe obesity and/or prevalence of T2D. On the other hand, LECT2 secretion from visceral adipose tissue may have an effect on the onset and progress of metabolic syndrome including NASH [24]. Yoo et al. demonstrated that circulating LECT2 levels were significantly associated with NAFLD (*p* < 0.001) and metabolic syndrome (*p* = 0.016) caused by mediating dyslipidemia and abdominal obesity [25]. On the other hand, LECT2 levels do not correctly reflect the inflammatory change and liver fibrosis required for the diagnosis of NASH. We can apply circulating LECT2 levels as a predictive marker of metabolic syndrome on the basis of previous relationships between LECT2 and MAFLD. Further detailed evaluations are warranted to apply LECT2 for distinguishing NASH in patients with MAFLD.

Lipid metabolism is another mechanism of progression from simple steatosis to NASH. First, fatty acid accumulation in hepatocytes causes mitochondrial damage that further aggravates fatty acid accumulation and results in liver inflammation [8, 22]. Second, arachidonic acid metabolism is accelerated by the overproduction of proinflammatory eicosanoids; then, prostaglandins, thromboxanes, and leukotrienes are produced by cyclooxygenase. We also demonstrated that a specific phosphatidylcholine (18:1e_20:4) involved in the arachidonic acid cascade changed significantly after LSG in severely obese Japanese patients with NASH [26]. Based on these results, further studies on SeP and LECT2 are needed to clarify the relationship between hepatokines and lipid metabolism.

Kim et al. demonstrated recently that adipose tissue LECT2 mRNA and serum LECT2 were higher in women with obesity and correlated significantly with parameters related to insulin resistance [24]. Moreover, immunochemical analysis of human tissues revealed that LECT2 is expressed in adipocytes [27]. Based on this background, comparing LECT2 mRNA and protein expression in both the liver and visceral fat tissue is mandatory to detect which is the dominant organ secreting LECT2. If both the liver and visceral fat tissue secrete LECT2 independently with the lipid accumulation of hepatocytes and visceral adipocytes, both T2D and NASH will ensue and worsen rapidly. Another study using human liver specimens and visceral adipocytes must be conducted to clarify this insight into patients with severe obesity. MS can be a strong therapeutic strategy to halt the overproduction of LECT2 if this insight is confirmed as the true phenomenon of metabolic syndrome.

When interpreting the results of the current study, some limitations must be considered. First, the number of patients we recruited was limited by the requirement for specific measuring kits for hepatokines. Second, the study itself involved only one institution and was retrospective, because a single institutional observational study might produce selection and confounding biases. Third, our results indicate only the early postoperative outcomes and changes associated with hepatokines. Long-term results of T2D remission and histopathological NASH improvement have received the most attention in the MS context; therefore, studying the long-term changes in hepatokines and the relationships between hepatokines and weight loss effects, metabolic parameters, and inflammatory parameters may yield new findings that could relate to another therapeutic target of these diseases [1, 19]. Ultimately, further studies with more participants and data from longer follow-up periods are warranted to evaluate the true effect of hepatokines on severely obese patients.

In conclusion, our study shows that hepatokines serve a crucial function in controlling obesity-related comorbidities such as T2D and NASH. In Japanese patients with both severe obesity and T2D, circulating levels of the diabetes-associated hepatokines, SeP, did not alter during the course of weight reduction, possibly biased by a reduction in glucose and insulin and metformin washout. However, the changes in SeP were correlated significantly with reductions in HbA1c and II after MS. On the other hand, circulating levels of the liver fat-associated hepatokines, LECT2, were closely correlated with the severity of NASH, including steatosis and inflammation. Reductions in LECT2 correlated significantly with reductions in weight and fat mass. These findings suggest the cause and consequence of hepatokines, contributing to the metabolic benefits of MS.

## Data Availability

The datasets generated and/or analyzed during the current study are available from the corresponding author on reasonable request. The data are not publicly available to protect the confidentiality of all patients as set by Japanese law; therefore, all data have been anonymized and are strictly protected from external network connection.

## References

[1] Misu H (2019). Identification of hepatokines involved in pathology of type 2 diabetes and obesity. Endocr J.

[2] Misu H (2018). Pathophysiological significance of hepatokine overproduction in type 2 diabetes. Diabetol Int.

[3] Meex RCR, Watt MJ (2017). Hepatokines: linking nonalcoholic fatty liver disease and insulin resistance. Nat Rev Endocrinol.

[4] Misu H, Takamura T, Takayama H, Hayashi H, Matsuzawa-Nagata N, Kurita S (2010). A liver-derived secretory protein, selenoprotein P, causes insulin resistance. Cell Metab.

[5] Misu H, Takayama H, Saito Y, Mita Y, Kikuchi A, Ishii KA (2017). Deficiency of the hepatokine selenoprotein P increases responsiveness to exercise in mice through upregulation of reactive oxygen species and AMP-activated protein kinase in muscle. Nat Med.

[6] Lan F, Misu H, Chikamoto K, Takayama H, Kikuchi A, Mohri K (2014). LECT2 functions as a hepatokine that links obesity to skeletal muscle insulin resistance. Diabetes.

[7] Chikamoto K, Misu H, Takayama H, Kikuchi A, Ishii KA, Lan F (2016). Rapid response of the steatosis-sensing hepatokine LECT2 during diet-induced weight cycling in mice. Biochem Biophys Res Commun.

[8] Takata N, Ishii KA, Takayama H, Nagashimada M, Kamoshita K, Tanaka T (2021). LECT2 as a hepatokine links liver steatosis to inflammation via activating tissue macrophages in NASH. Sci Rep.

[9] Sasaki A, Yokote K, Naitoh T, Fujikura J, Hayashi K, Hirota Y (2021). Metabolic surgery in treatment of obese Japanese patients with type 2 diabetes: a joint consensus statement from the Japanese Society for Treatment of Obesity, the Japan Diabetes Society, and the Japan Society for the Study of Obesity. Diabetol Int.

[10] Tanaka M, Saito Y, Misu H, Kato S, Kita Y, Takeshita Y (2016). Development of a Sol Particle Homogeneous Immunoassay for Measuring Full-Length Selenoprotein P in Human Serum. J Clin Lab Anal.

[11] Angulo P, Hui JM, Marchesini G, Bugianesi E, George J, Farrell GC (2007). The NAFLD fibrosis score: a noninvasive system that identifies liver fibrosis in patients with NAFLD. Hepatology.

[12] Brunt EM, Janney CG, Di Bisceglie AM, Neuschwander-Tetri BA, Bacon BR (1999). Nonalcoholic steatohepatitis: a proposal for grading and staging the histological lesions. Am J Gastroenterol.

[13] Sakamoto M, Tsujikawa H, Effendi K, Ojima H, Harada K, Zen Y (2017). Pathological findings of nonalcoholic steatohepatitis and nonalcoholic fatty liver disease. Pathol Int.

[14] Stefan N, Schick F, Birkenfeld AL, Häring HU, White MF (2023). The role of hepatokines in NAFLD. Cell Metab.

[15] Zhang Z, Yuan Y, Hu L, Tang J, Meng Z, Dai L (2023). ANGPTL8 accelerates liver fibrosis mediated by HFD-induced inflammatory activity via LILRB2/ERK signaling pathways. J Adv Res.

[16] Liu XH, Qi LW, Alolga RN, Liu Q (2022). Implication of the hepatokine, fibrinogen-like protein 1 in liver diseases, metabolic disorders and cancer: The need to harness its full potential. Int J Biol Sci.

[17] Takayama H, Misu H, Iwama H, Chikamoto K, Saito Y, Murao K (2014). Metformin suppresses expression of the selenoprotein P gene via an AMP-activated kinase (AMPK)/FoxO3a pathway in H4IIEC3 hepatocytes. J Biol Chem.

[18] Eslam M, Newsome PN, Sarin SK, Anstee QM, Targher G, Romero-Gomez M (2020). A new definition for metabolic dysfunction-associated fatty liver disease: An international expert consensus statement. J Hepatol.

[19] Nikai H, Sasaki A, Umemura A, Takahashi N, Nitta H, Akasaka R (2021). Predictive scoring system for advanced liver fibrosis in Japanese patients with severe obesity. Surg Today.

[20] Kakisaka K, Sasaki A, Umemura A, Nikai H, Suzuki Y, Nishiya M (2021). High frequency and long persistency of ballooning hepatocyte were associated with glucose intolerance in patients with severe obesity. Sci Rep.

[21] Sasaki A, Nitta H, Otsuka K, Umemura A, Baba S, Obuchi T (2014). Bariatric surgery and non-alcoholic fatty liver disease: current and potential future treatments. Front Endocrinol (Lausanne).

[22] Gwag T, Reddy Mooli RG, Li D, Lee S, Lee EY, Wang S (2020). Macrophage-derived thrombospondin 1 promotes obesity-associated non-alcoholic fatty liver disease. JHEP Rep.

[23] Li M, Liu L, Kang Y, Huang S, Xiao Y (2023). Circulating THBS1: A Risk Factor for Nonalcoholic Fatty Liver Disease in Obese Children. Ann Nutr Metab.

[24] Kim J, Lee SK, Kim D, Lee E, Park CY, Choe H (2022). Adipose tissue LECT2 expression is associated with obesity and insulin resistance in Korean women. Obesity (Silver Spring).

[25] Yoo HJ, Hwang SY, Choi JH, Lee HJ, Chung HS, Seo JA (2017). Association of leukocyte cell-derived chemotaxin 2 (LECT2) with NAFLD, metabolic syndrome, and atherosclerosis. PLoS ONE.

[26] Takahashi N, Sasaki A, Umemura A, Sugai T, Kakisaka K, Ishigaki Y (2022). Identification of a Fatty Acid for Diagnosing Non-Alcoholic Steatohepatitis in Patients with Severe Obesity Undergoing Metabolic Surgery. Biomedicines.

[27] Nagai H, Hamada T, Uchida T, Yamagoe S, Suzuki K (1998). Systemic expression of a newly recognized protein, LECT2, in the human body. Pathol Int.

